# Preterm births prevalence during the COVID-19 pandemic in Brazil: results from the national database

**DOI:** 10.1038/s41598-023-37871-x

**Published:** 2023-09-04

**Authors:** Charles M’poca Charles, Luiz Alves Souza Neto, Camila Ferreira Soares, Tacildo Souza Araújo, Cristiano Torezzan, Everton Emanuel Campos Lima, Aline Munezero, Luis Bahamondes, Renato Teixeira Souza, Maria Laura Costa, José Guilherme Cecatti, Rodolfo Carvalho Pacagnella

**Affiliations:** 1 Department of Obstetrics and Gynecology, University of Campinas, Campinas, SP Brazil; 2 Provincial Health Administration, DPS Manica, Chimoio, Mozambique; 3 Institute of Mathematics, Statistics and Scientific Computing (IMEEC), University of Campinas, Campinas, SP Brazil; 4 School of Applied Sciences (FCA), University of Campinas, Campinas, SP Brazil; 5 College of Philosophy and Human Sciences (IFCH), University of Campinas, Campinas, SP Brazil; 6 Center for Population Studies (NEPO), University of Campinas, Campinas, SP Brazil; 7 Campinas Reproductive Health Research Center (CEMICAMP), Campinas, Brasil

**Keywords:** Medical research, Epidemiology

## Abstract

The SARS-CoV-2 (COVID-19) pandemic impacted the health systems between and within countries, and in the course of the pandemic sexual and reproductive health services were the most disrupted. Findings from high-income settings have reported significant changes in preterm birth prevalence during the pandemic period. To understand the possible effects of the COVID-19 pandemic on preterm birth numbers at the Brazilian national level. We compare the number of preterm deliveries during the COVID-19 pandemic period (2020 and 2021) with previous years. We conducted a population-based cross-sectional study taking the period from January 2017 to December 2021 to account. We use individual-level live births data from the Brazilian Live Birth Information System (SINASC), and we estimate the odds ratio (OR) of preterm deliveries using propensity score weighting analysis in Brazil and its regions. During the study period (from 2017 to 2021), about 2.7 million live births were recorded per year, and the missing value for gestational age at delivery was less than 1.5%. The preterm birth prevalence slightly increased during the COVID-19 pandemic compared to the pre-pandemic period (11.32% in 2021 vs 11.09% in 2019, p-value < 0.0001). After adjusting for sociodemographic variables, the OR of preterm births in Brazil has significantly increased, 4% in 2020 (OR: 1.04 [1.03–1.05] 95% CI, p-value < 0.001), and 2% in 2021(OR: 1.02 [1.01–1.03] 95% CI, p-value < 0.001), compared to 2019. At the regional level, the preterm birth pattern in the South, Southeast and Northeast regions show a similar pattern. The highest odds ratio was observed in the South region (2020 vs 2019, OR: 1.07 [1.05–1.10] 95% CI; 2021 vs 2019, OR: 1.03 [1.01–1.06] 95% CI). However, we also observed a significant reduction in the ORs of preterm births in the northern region during the COVID-19 pandemic (2020 vs 2019, OR: 0.96 [0.94–0.98] 95% CI) and (2021 vs 2019, OR: 0.97 [0.95–0.99] 95% CI). Our analysis shows that the pandemic has increased regional variation in the number of preterm births in Brazil in 2020 and 2021 compared to the pre-pandemic years.

## Introduction

The COVID-19 pandemic impacted Latin American countries as hard as in more developed locations. Due to the sanitary crises, we saw in many countries disrupting health systems, and as consequence, mortality exceeded its usual numbers and life expectancy reductions in many countries^1^. In addition to controversial health policies, conflicting messages and long-time central government resistance to implementing population mobility restrictions^2^, Brazil was one of the most affected countries by COVID-19 worldwide. Pregnant women were also a risk group, as maternal mortality skyrocketed during the pandemic^3^.

Maternal mortality is also an important proxy for the quality of country's health services. Another obstetric condition that is sensitive to suboptimal clinical care is preterm birth^4^. Studying preterm birth is important because it is the primary cause of neonatal deaths, and its prevalence is rising in most low- and middle-income countries, despite many efforts to revert it^5,6^. While several risk factors have been well-established, the key factor responsible for preterm deliveries remains unknown in half of the cases^7^. Since the onset of the pandemic, several studies have identified an association between COVID-19 infection and adverse perinatal outcomes, such as stillbirths and premature deliveries^8–11^. These findings are also contradictory because some analyses indicate that the number of preterm deliveries increased during the pandemic, while other studies suggested a reduction in such types of pregnancies^11^.

One of the most important underlying mechanisms for preterm birth is the inflammatory condition. The systemic inflammation may trigger cervical effacement and uterine contraction through increasing prostaglandins^12^. The SARS-CoV-2 infection is a systemic inflammatory disease; therefore, we may argue that it could lead to preterm birth. For example, among women with SARS-CoV-2 pneumonia, empirical evidence shows an increased preterm birth rate^13^. However, the infection itself may not represent the whole mechanism related to preterm delivery.

In addition, we may also argue that changes in individual behavior are associated with lockdown and other population restrictions policies, implemented to mitigate SARS-CoV-2 dissemination, and that may have influenced to some extent the number of preterm births. As an example, an Australian study showed a lower risk of preterm birth in pregnant women during lockdowns in comparison to those born before the pandemic^14^. Other studies also indicate a decrease in preterm birth rates^15,16^, although the same empirical evidence was not corroborated elsewhere that fail to identify differences in the number of preterm pregnancies^17^.

Despite inconclusive findings, there is a consensus that the COVID-19 pandemic period brought many challenges to the country’s health systems, and there is still scarce information about its real consequences on perinatal health while considering low- and middle-income countries. Therefore, in this study, we aim to assess the changes in preterm birth counts in Brazil and its regions, by comparing the number of preterm deliveries during the pandemic (2020 and 2021) and pre-pandemic periods (2017, 2018 and 2019).

## Methods

We performed extensive use of the publicly available microdata of live birth, collected by the Brazilian Ministry of Health, and launched by the Brazilian Live Birth Information System (SINASC in Portuguese)^18^. The SINASC is an e-birth registration system developed by the Department of Informatics of the National Unified Health System (DATASUS). This system was implemented in 1990. The data are collected routinely immediately after each birth through a standardized document (declaration of live births), which was updated in 2010 to ensure a better quality of the information recorded^19^. The updated version included many important variables for the study of preterm birth, such as sociodemographic and obstetric variables.

The data was downloaded (as of August 12, 2022), and updated (as of April 15, 2023) from http://svs.aids.gov.br/dantps/cgiae/sinasc/, and we consider all live births equal or superior to 22 weeks, from January 2017 to December 2021. We extracted individual-level data regarding gestational age at birth, maternal age, marital status, ethnicity, schooling (as a proxy of women's income), parity, gravidity, mode of delivery, region and federal state of residence, number of living children, number of antenatal care (ANC) visits, and newborn's weight and sex. These variables are available in the SINASC for each birth, and they are highly associated with preterm delivery. We did not exclude multiple pregnancies and neonates with congenital anomalies for the analysis. All categorical variables were converted to binary dummies by using the one-hot encoding procedure. Less than 1.5% of the data had missing or unknown information. Notwithstanding, SINASC data quality has recently shown enormous improvement. Of course, while considering more disaggregated geographical levels, the data may still need some adjustments and corrections. For this study, we work with Brazil and great regions, and that reduces significantly defective concerns such as under-registration of birth counts; as in Brazil and its regions, the rate of underreported data is generally less than 1%, except in the North and Northeast regions where the rate is about^20,21^ 1.7%*.* In addition, Castanheira and Kohler considered inadequate to apply any correction method to birth registrations, given the recent fertility dynamics in the country^22^. Lima et al.^23^ also show that recent SINASC information does not require data corrections at lesser disaggregated levels, such as Federal States and great regions*.* However, we acknowledge that the unprecedented burden on the health system during the pandemic may have influenced the data quality. The study protocol was published elsewhere^24^.

To reduce the influence of past trends in prematurity prevalence, we restrict our preterm birth analysis to pairwise years comparison, initiating from 2017 until 2021. We did not include data before 2017 to avoid the influence of the Zika virus outbreak (between 2015 and 2016) on birth counts and overall fertility^25^. We created four stacked datasets (2017–2018; 2018–2019; 2019–2020; 2019–2021) and we added, for each dataset, two dichotomous variables of interest: one to indicate whether the birth was preterm (y = 1) and (y = 0) otherwise, and another measure indicates the period, i.e. the current year in the dataset (z = 0) vs. the following year (z = 1). This last variable is useful for identifying the control group (preterm births occurring in years before COVID-19) and the treatment group (preterm deliveries occurring during the pandemic).

### Statistic model

Our analysis was based on a quasi-experimental approach using a Propensity Score Weighting (PSW) method^26,27^. PSW was designed to control for selection bias in non-experimental studies, for which it is desirable to assess the average effect of some variable that emulates a control/treatment process. Propensity scores are used to match untreated versus treated individuals, understanding that there is a probability of these last being exposed to a certain stimulus or intervention^28^.

As the first step, a multiple logistic regression analysis was used to fit the binary control variable (z) as a function of the mother’s and obstetrics’ characteristics: age, ethnicity/skin colour, schooling, parity, mode of delivery, number of previous children, marital status, number of antenatal care visits and new-born weight. With the regression estimates, we extracted a vector (e) that gives the probability of treatment assignment to a random individual conditioned to a given set of covariates (x), i.e. e(x) = P (z = 1| x).

**Figure 1:**
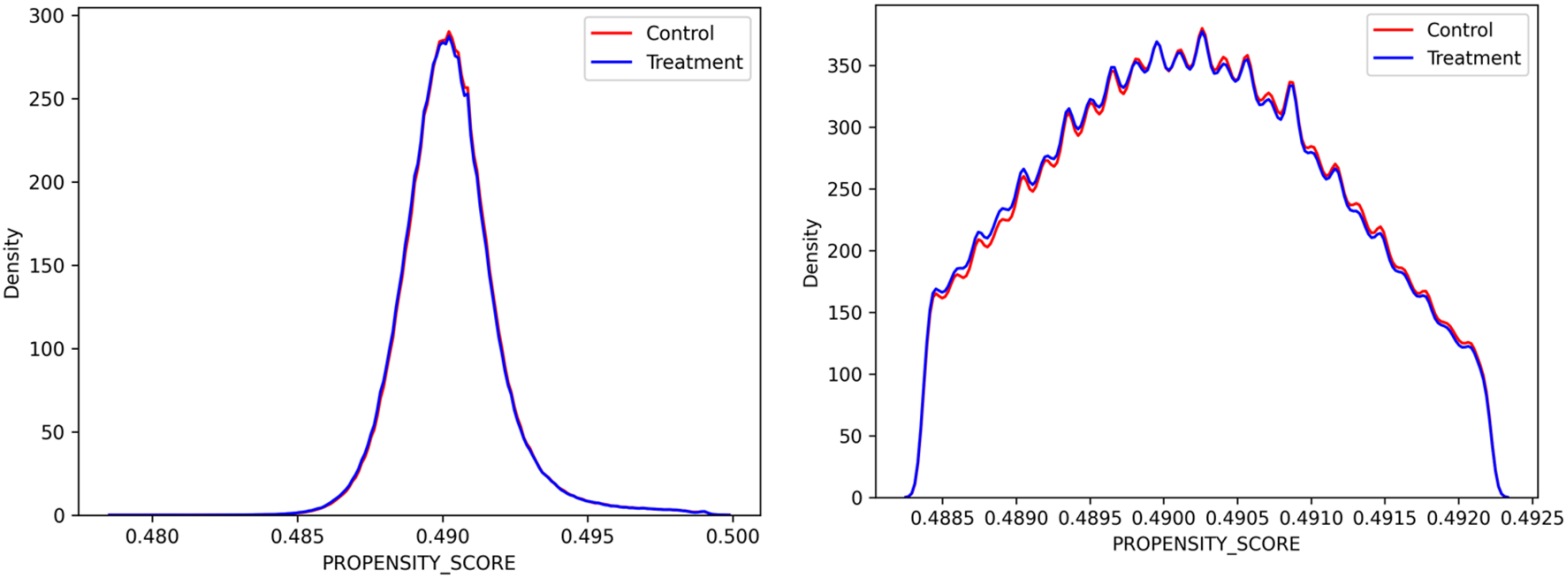
Example of Kernel density estimate (KDE) plot for the Propensity Score referring to births in Brazil in the years 2019 and 2020. *Source* Brazilian Live Birth Information System (SINASC) (2023).

The vector (e) is called the Propensity Score, and it was used to control for selection bias and to derive the weights of a second regression model. The control was made by pruning samples corresponding to the tails of the Propensity Score vector, to keep only samples that can be considered comparable to each other. Figure 1 shows the kernel density estimate (KDE) plot for the Propensity Score referring to births in Brazil in the years 2019 and 2020, before (a) and after (b) a 10% pruning of each tail. In this example, 80% of the original dataset was selected for the final phase of the analysis.

The set of weights was estimated as follows: for the individuals in the treatment group, w = 1/e(x), and for the individuals in the control group w = 1/(1-e(x)). As a final step, we estimate a new regression, fitting the outcome of interest (preterm birth) controlled by the covariates and using the propensity scores as weights.

## Results

About 2.7 million live births were recorded annually from 2017 to 2021^18^. In Table 1, we present the percentage share of clinical and socioeconomic mother’s characteristics, comparing the last three years of our analysis, 2019 until 2021.

**Table 1:** Descriptive statistics for Brazil live births 2019, 2020 and 2021.

Variables	*2019*		*2020*		*2021*	
	*%*	N 2,849,146	*%*	N 2,730,145	*%*	N 2,672,046
Gestational age at delivery						
Term	87.64	2,497,002	87.40	2,386,104	87.28	2,332,351
Preterm	11.09	315,831	11.31	308,702	11.32	302,677
Not stated/unknown	1.27	36,313	1.30	35,339	1.38	37,018
Mother’s age						
< 19	10.38	295,832	9.88	269,839	9.62	257,026
19–34	69.84	1,989,893	69.90	1,908,361	69.93	1,868,652
> 34	19.78	563,421	20.22	551,945	20.45	546,368
Parity						
Primiparous	37.54	1,069,586	37.04	1,011,438	36.79	983,071
Multiparous	62.46	1,779,560	62.95	1,718,707	63.20	1,688,975
Mode of delivery						
Vaginal	43.63	1,243,104	42.69	1,165,641	42.89	1,145,970
Caesarean	56.3	1,604,189	57.22	1,562,282	57.04	1,524,013
Not stated/Unknown	0.07	1,853	0.08	2,222	0.08	2,063
Newborn Sex						
Male	51.15	1,457,226	51.20	1,398,043	51.16	1,367,051
Female	48.84	1,391,486	48.78	1,331,658	48.82	1,304,590
Not stated/unknown	0.02	434	0.02	444	0.02	405
Race/color						
White	33.85	964,557	32.26	908,547	32.47	867,657
Black	6.19	176,224	6.34	179,416	6.81	181,875
Asian	0.45	12,738	0.44	12,309	0.45	12,106
Brown	55.96	1,594,267	57.06	1,533,251	56.75	1,516,269
Indigenous	0.93	26,373	0.91	25,741	1.06	28,216
Not stated/Unknown	2.63	74,987	3	70,881	2.47	65,923
Mother’s schooling						
0 to 7 years	16.22	462,063	15.36	431,144	14.21	379,799
8 to 11 years	61.36	1,748,186	62.23	1,698,877	62.62	1,673,570
12 and more	21.27	606,145	21.12	589,807	21.48	583,779
Not stated/unknown	1.15	32,752	1.31	29,178	1.30	34,898
Mother’s marital status						
Single	45.14	1,285,998	47.02	1,283,754	48.39	1,292,963
Married/Cohabit	52.39	1,492,765	50.34	1,374,363	48.76	1,302,820
Widow	0.16	4,693	0.17	4,603	0.19	4,978
Divorced	1.36	38,748	1.45	39,619	1.48	39,576
Not stated/Unknown	0.95	26,942	1.02	27,806	1.19	31,709
Type of pregnancy						
Single	97.76	2,785,200	97.74	2,668,636	97.72	2,611,194
Twin	2.13	60,61	2.11	57,846	2.14	57,061
Triplet and more	0.05	1,467	0.05	1,262	0.05	1,319
Not stated/Unknown	0.07	1,869	0.08	2,401	0.09	2,472
Number of antenatal visits						
None	1.52	43,406	1.73	47,276	1.84	49,085
1 to 3	5.35	152,483	6.04	164,943	5.34	142,687
4 to 6	20.26	577,17	20.70	565,211	19.15	511,652
7 and more	72.43	2,063,669	71.01	1,938,920	73.14	1,954,282
Not stated/Unknown	0.44	12,418	0.50	13,795	0.54	14,430

*Source* Brazilian Live Birth Information System (SINASC) (2023).

Overall, in Brazil, the preterm birth counts were around 11%, and this number did not change much compared to pre-pandemic years, in this case, 2019. Also, in terms of the mother’s characteristics, we did not identify considerable changes in the last three years of our analysis.

In Table 2, we show the results of the multiple regression analysis using PSW for Brazil, by pairwise year comparison. We were interested to see if the pandemic (treatment period, 2020, and 2021) somehow affected the chances of preterm birth counts in the country. Our results show that the Odds Ratio (ORs) of preterm births in Brazil has increased by 4% in 2020 (95% CI 1.03–1.05), and 2% (95% CI 1.01–1.03) in 2021, compared to 2019 after controlling for other sociodemographic variables.

**Table 2:** Logistic regression analysis using Propensity Score Weighting for preterm birth in Brazil 2017–2021.

	Odds ratio (95% CI)			
	2017–2018	2018–2019	2019–2020	2019–2021
Intercept	157.97*** (152.54–163.60)	31.54*** (29.90–33.27)	33.12*** (31.39–34.95)	35.41*** (33.56–37.36)
Year	1.01** (1.00–1.02)	1.00 (0.99–1.1)	1.04*** (1.03–1.05)	1.02*** (1.01–1.03)
Weight	0.99*** (0.99–0.99)	0.99*** (0.99–0.99)	0.99*** (0.99–0.99)	0.99*** (0.99–0.99)
Mother’s age				
19–34	REF	REF	REF	REF
< 19	1.10*** (1.11–1.12)	1.20*** (1.19–1.21)	1.19*** (1.18–1.21)	1.20*** (1.19–1.22)
> 34	1.24*** (1.23–1.25)	1.23*** (1.22–1.24)	1.26*** (1.25–1.27)	1.26*** (1.25–1.27)
Multiparous	1.11*** (1.10–1.12)	1.17*** (1.16–1.18)	1.19*** (1.18–1.20)	1.20*** (1.19–1.21)
Caesarean delivery	1.08*** (1.07–1.09)	1.08*** (1.07–1.09)	1.09*** (1.08–1.10)	1.10***(1.09–1.11)
Sex				
Female	Ref	Ref	Ref	Ref
Unknown	1.74*** (1.40–2.18)	2.66*** (1.93–3.67)	2.79*** (2.03–3.84)	2.55*** (1.83–3.55)
Male	1.38*** (1.37–1.39)	1.38*** (1.37–1.39)	1.39*** (1.38–1.40)	1.40*** (1.39–1.41)
Race/colour				
White	Ref	Ref	Ref	Ref
Black	0.90*** (0.88–0.92)	0.90*** (0.89–0.91)	0.90*** (0.88–0.91)	0.89*** (0.88–0.91)
Asian	0.90 (0.85–0.95)	0.94 (0.89–0.98)	0.99 (0.94–1.05)	0.97 (0.92–1.03)
Brown	0.98*** (0.97–0.99)	0.99 (0.98–1.00)	1.03*** (1.02–1.04)	1.04*** (1.03–1.05)
Indigenous	1.05*** (1.01–1.10)	1.10*** (1.07–1.14)	1.50*** (1.44–1.54)	1.60*** (1.57–1.63)
Mother’s schooling				
12 and more	ref	ref	ref	ref
8 to 11 years	0.96 (0.95–0.97)	1.04*** (1.03–1.05)	1.05*** (1.04–1.06)	1.05***(1.03–1.06)
0 to 7 years	1.00 (0.99–1.01)	1.14*** (1.13–1.16)	1.15*** (1.13–1.16)	1.14*** (1.12–1.16)
Mother’s marital status				
Single	0.95*** (0.94–0.96)	0.97*** (0.96–0.98)	0.93*** (0.92–0.94)	0.95*** (0.94–0.96)
Married/Cohabit	ref	ref	ref	ref
Widow	1.00 (0.92–1.98)	0.99 (0.89–1.09)	0.96 (0.88–1.06)	1.05 (0.96–1.15)
Type of pregnancy				
Single	ref	ref	ref	ref
Twin	2.91*** (2.86–2.96)	3.44*** (2.36–3.52)	3.44*** (3.36–3.52)	3.49*** (3.42–3.58)
Triplet and more	11.70*** (9.55–14.34)	4.36*** (2.68–7.09)	4.56*** (2.86–7.28)	5.24*** (3.29–8.34)
Number of antenatal visits				
None	ref	ref	ref	ref
1 to 3	1.52*** (1.48–1.57)	1.59*** (1.53–1.64)	1.50*** (1.45–1.55)	1.44*** (1.39–1.49)
4 to 6	1.26*** (1.23–1.30)	1.32*** (1.28–1.37)	1.28*** (1.24–1.33)	1.24***(1.20–1.29)
7 and more	0.66*** (0.64–0.67)	0.70*** (0.68–0.72)	0.70*** (0.67–0.72)	0.67*** (0.65–0.69)
N	4,379,012	4,363,507	4,218,951	4,169,362

*Source* Brazilian Live Birth Information System (SINASC) (2023).

Significance level p < 0.05*, p < 0.01** and p < 0.001***.

In addition, the pairwise comparison for the period 2017 to 2019 shows small or non-significant changes in the ORs of preterm births, and the odds ratios of preterm pregnancies fluctuated between values of below and above 1%. This means that during the pandemic the chances of preterm deliveries have increased somewhat to two and four per cent.

**Figure 2:**
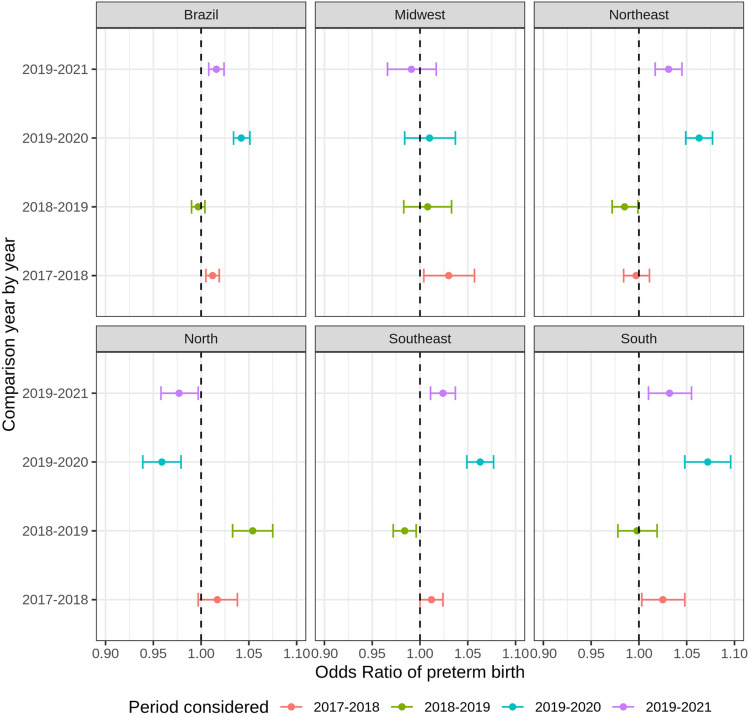
Odds-ratios of preterm birth for Brazil and its regions 2017 to 2021. *Source* Brazilian Live Birth Information System (SINASC) (2023).

In Fig. 2, we bring the odds ratios for Brazil and its regions. These estimates are based on complete models, controlled by the same variables described in Table 2. Across regions, the odds ratios of preterm births showed a small decline or even stalled values between the pre-pandemic periods of 2017–2019, seen especially in the South and Midwest regions of Brazil. However, while we consider the pandemic period effect (2019 vs. 2020, and 2019 vs. 2021), the chances of preterm pregnancies increased again. In the Southeast and the less developed Brazilian region of the Northeast, for example, there was a small decrease in the odds ratios between 2017 and 2019, but during the pandemic period, the chances of preterm births increased even more, especially in the Northeastern part of Brazil. The Northern region was the only location that had a reduction in the odds ratios of preterm births during the COVID-19 pandemic period (2020 and 2021). These results may also indicate that the effect of the pandemic on the prevalence of preterm births was uneven across subnational areas of the country.

**Figure 3:**
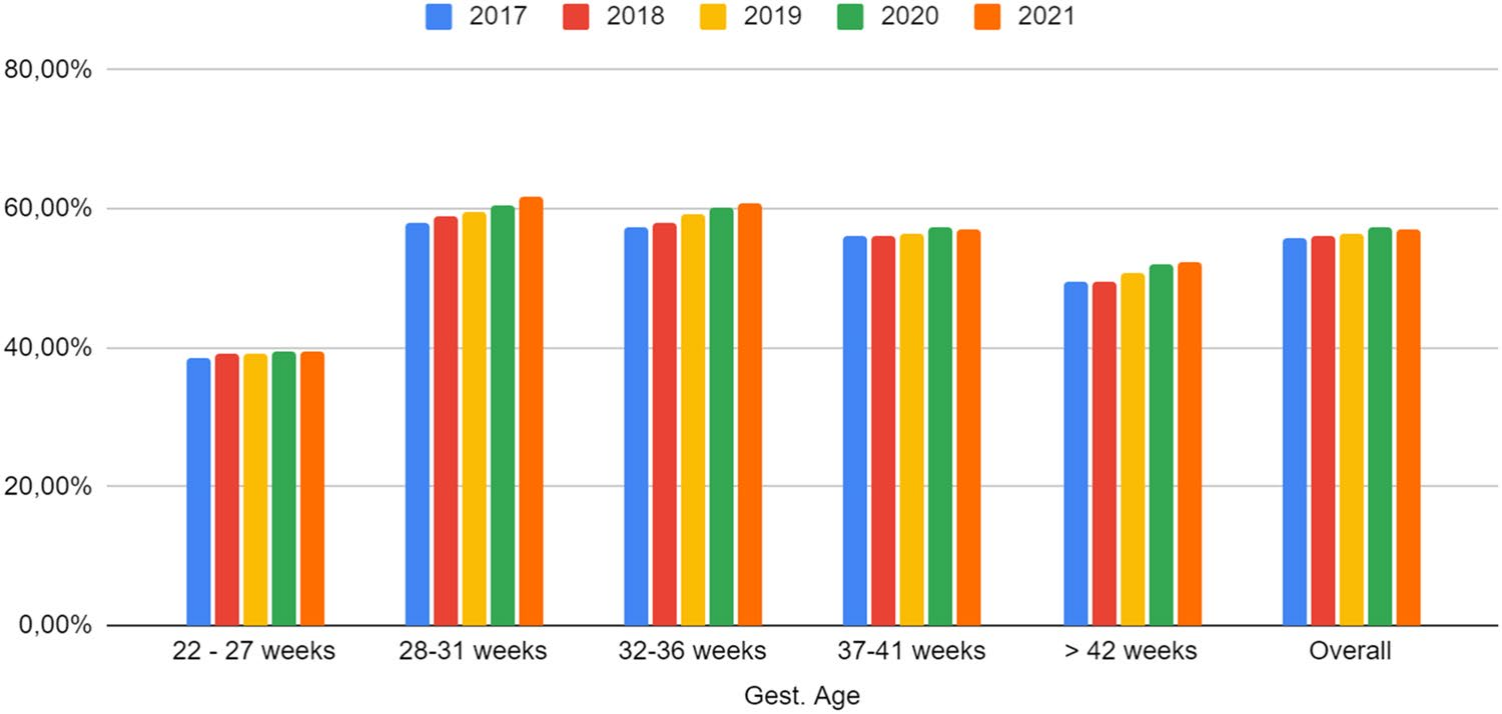
Caesarean delivery rate for categories of gestational ages in Brazil (2017 to 2021). *Source* Brazilian Live Birth Information System (SINASC) (2023).

**Figure 4:**
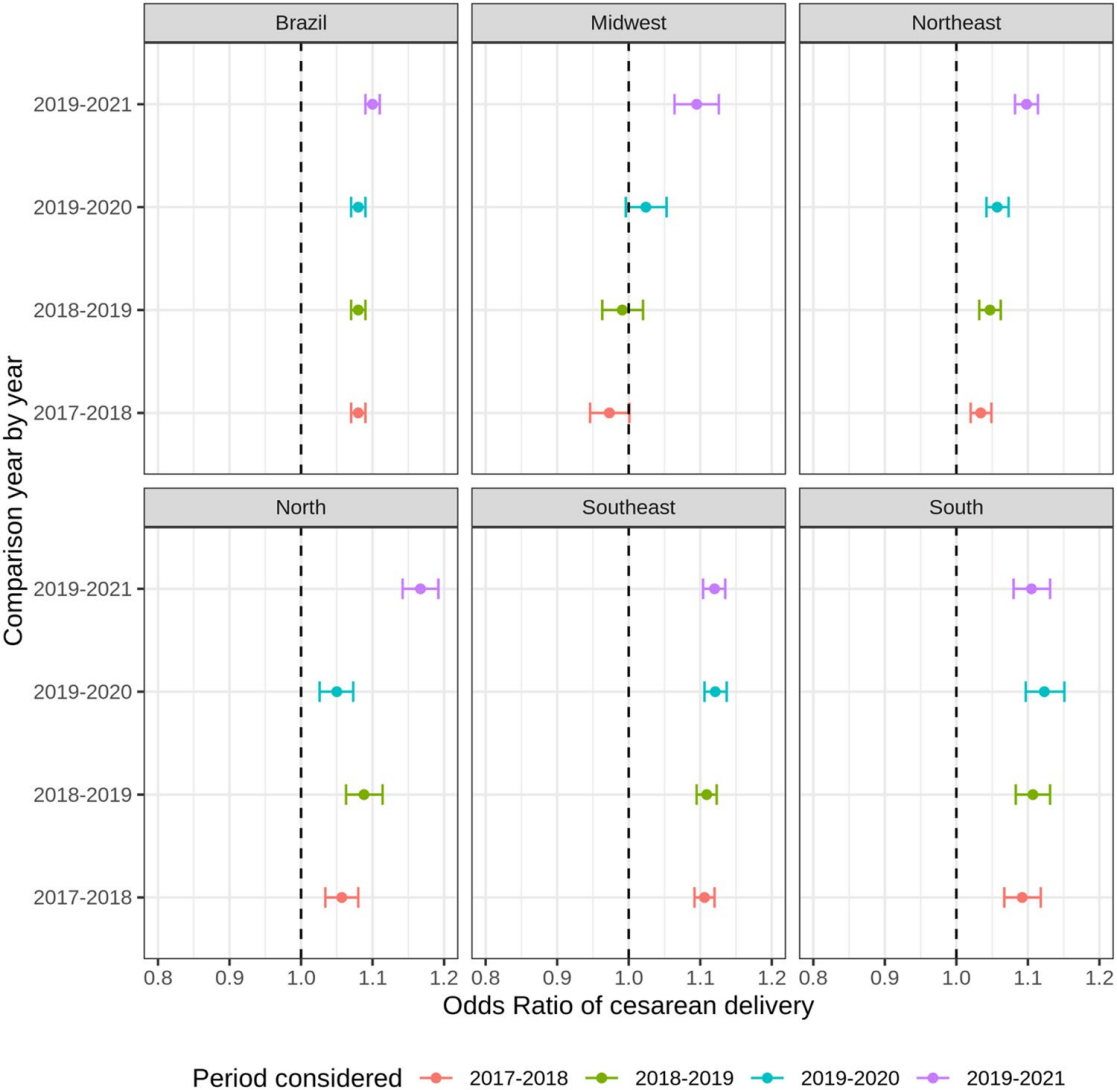
Odds-ratio of caesarean delivery among preterm babies in Brazil and regions (2017 to 2020). *Source* Brazilian Live Birth Information System (SINASC) (2023).

Our finding showed a significant change in caesarean delivery rate during the pandemic period compared to the previous period (OR 1.09 [1.08–1.10] in 2020, and OR 1.10 [1.09–1.11] in 2021), Table 2. The analysis of the mode of delivery by gestational age, for the pairwise comparison of 2019 and 2020, showed a trend of increasing caesarean delivery in all gestational age groups. Moreover, preterm babies had a higher risk of being delivered by caesarean in 2020 and 2021 compared to the previous years. This pattern was also observed in the South, Southeast, and Northeast regions, Figs. 3 and 4.

## Discussion

Using Ministry of Health data, we assessed the odds ratios of preterm births in Brazil and its regions, before and during the pandemic. Our results indicated that, during the pandemic years of 2020 and 2021, preterm births have significantly increased as compared to pre-pandemic periods. This increase was not homogeneous across the country, and in certain regions, the pandemic has disrupted previous decline patterns or even accelerated the past trend of preterm deliveries growing; as observed in Northeastern, Southeastern, and Southern regions.

The ethnicity (indigenous women), low level of education, low number of antenatal care visits, and multiparity, extreme maternal ages, were associated with an increased risk of PTB. These data are similar to the findings of other population-based studies^29,30^.

Finding from individual studies and systematic review has suggested a global reduction in ANC clinic visits, maternity healthcare-seeking, and unscheduled care visits^31^; the same pattern was also observed in Brazil, where the quality of ANC was low (only 35.8% of the study participants had adequate), In addition, the risk of inadequate ANC was higher among pregnant women with black/brown skin colour and multiparous when compared to their contra part^32^. These factors potentially contributed to worsening pregnancy outcomes (including the preterm birth rate), even for married/cohabiting women.

During the COVID-19 access to the Internet and DICT (Digital Information and Communication Technologies) was heterogeneous within the Brazilian regions, and municipality, public and private health systems. And, to the best of our knowledge, data regarding the coverage of virtual or remote antenatal care were not available in the database [SINASC], and the ANC visits are not desegregated by the mode of consultation [remote vs in-person]), therefore, we have not considered this variable in our analysis.

The pandemic brought the attention of health experts and demographers that took the time to understand how COVID-19 could affect birth counts and, for instance, the chances of preterm deliveries in the country. Brazil is a country that suffered excessive mortality due to the COVID-19 pandemic^2,33^, as well as health facilities also were stressed by the high number of COVID-19 cases, and many services could not be properly provided by health units^34^. This exogenous sanitary problem might have also affected women's antenatal care, especially among those that require more attention from public health services, i.e., mothers from low socioeconomic strata. Uncertainty and economic restrictions caused by the pandemic context may also play an important role in reproduction^35^, and compromise pregnancy and antenatal care in Brazil.

Among Brazilian regions, the Northeast requires special attention because this is a region marked by historically lower socioeconomic development that could be in turn associated with restricted health services access^36,37^, and the lack of strategy to mitigate the impact of the pandemic at different governmental levels^3,33^. Notwithstanding, the COVID-19 pandemic brought an enormous burden to Brazil's Northern and Northeastern regions and revealed a sudden disruption of health care services^38,39^. These setbacks might in turn affect the preterm birth rate.

Our findings differ from other studies that indicated a reduction in preterm deliveries during the COVID-19 pandemic^10,14,40–44^. This could be partly explained by the measures applied to face the pandemic, which was uncoordinatedly implemented in Brazil^36^. Regional inequality in health services access and the slow degree of responsiveness of the Brazilian National Health System could have played a role in the unequal pandemic effects on preterm births across Brazilian regions. As previous studies indicate, less sub-national inequality is seen in high-income countries, recognized by strict lockdown policies and with developed health services according to the needs posed by the pandemic. Moreover, Brazil had more severe cases; one out of seven maternities had intensive unit beds, therefore resulting in the phase three delay—concerning receiving proper diagnosis and timely treatment^1,45,46^.

However, our findings suggested a different pattern of preterm birth rate in the Northern region. In the Northern region of Brazil, more than two–thirds of pregnant women did not attend antenatal care, and higher excess mortality (especially in Manaus city), which might have caused severe perinatal outcomes (miscarriage and fetal death)^47,48^.

Our study suggested an increased rate of caesarean delivery among preterm babies in 2020, and 2021 compared to previous periods. Therefore, we may speculate that the increased risk of PTB in 2020, and 2021 may be related to non-spontaneous (provider-initiated) preterm birth^49,50^.

It is important to mention that we concentrate our analysis and interpretations on the year's effect only (comparison between control versus treatment, or pre-pandemic vs. pandemic period), and we do not get into detail about the other control variables, despite the models have shown important differences in preterm pregnancies among distinguished demographic and socioeconomic groups.

This study has some strengths and limitations. Our data covers the entire population of live births in Brazil, with information at the individual level^20^. The analysis of the different geographic regions allowed us to picture preterm birth developments in a country recognized for its regional inequality. The main limitation is related to the study design, which does not allow us to infer causality but only refers to the association between the pandemic and preterm births. We also did not assess the direct impact of COVID-19 on the occurrence of preterm births, and we considered the years 2020 and 2021 as risk factors that caused changes (from social, economic, and epidemiological order) brought by the pandemic onset. Likewise, our model did not include all variables associated with preterm birth, for example, human development index, availability and access to health services before and during the COVID-19 pandemic, cigarette smoking, BMI, maternal income, unemployment, maternal underlying medical conditions, and maternal infection (vector-borne diseases, urinary tract, genital, and respiratory infection [including COVID-19]). We did not assess the prevalence of fetal deaths and the abortion rates. But we recognize that these outcomes could have increased in situations of reduced access to adequate health services, impacting Brazil’s birth rates.

Although we did not see an expressive increase of preterm births, we still argue that the disruption of sexual and reproductive health services may have influenced pregnancy outcomes. Therefore, monitoring the preterm birth rate might be an essential strategy for assessing the quality of maternal and perinatal care and might help providers and policymakers to develop strategies to mitigate the problem.

## Data Availability

The datasets analysed during the current study are publicly available from the Brazilian Live Birth Information System (SINASC) http://svs.aids.gov.br/dantps/cgiae/sinasc/.
